# Synthesis and Characterization of a 1,2,4-Diazarsolide Anion

**DOI:** 10.1021/acs.organomet.4c00476

**Published:** 2024-12-30

**Authors:** William D. Jobbins, Bono van IJzendoorn, Meera Mehta

**Affiliations:** † Department of Chemistry, University of Manchester, Oxford Rd, Manchester, M13 9PL, United Kingdom; ‡ Department of Chemistry, University of Oxford, 12 Mansfield Road, Oxford, OX1 3TA, United Kingdom

## Abstract

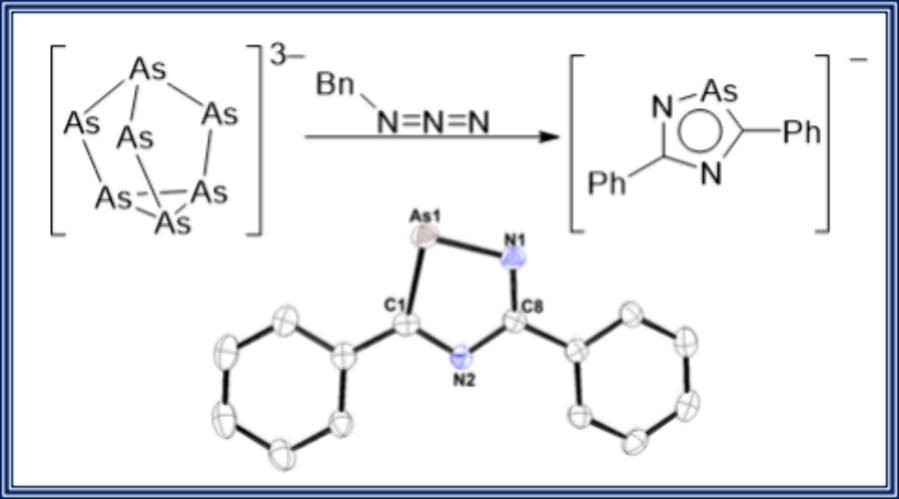

Cyclopentadienyl anions ([Cp]^−^) are pervasive ligands in coordination chemistry. In contrast, heavy-element derivatives of these ligands, particularly those that feature arsenic, are not as well developed. In this work, a new arsenic-based heterocycle with a structure analogous to [Cp]^−^ is presented. Reaction of K_3_As_7_ with benzyl azide (BnN_3_) leads to fragmentation of the [As_7_]^3–^ core and C–H activation of two [BnN] units to give the five-membered arsenic heterocycle [As(NC(Ph))_2_]^−^. This arsenic heterocycle was studied by nuclear magnetic resonance and UV–vis spectroscopy, X-ray diffraction, and its electronic structure was investigated computationally.

## Introduction

**Figure 1:**
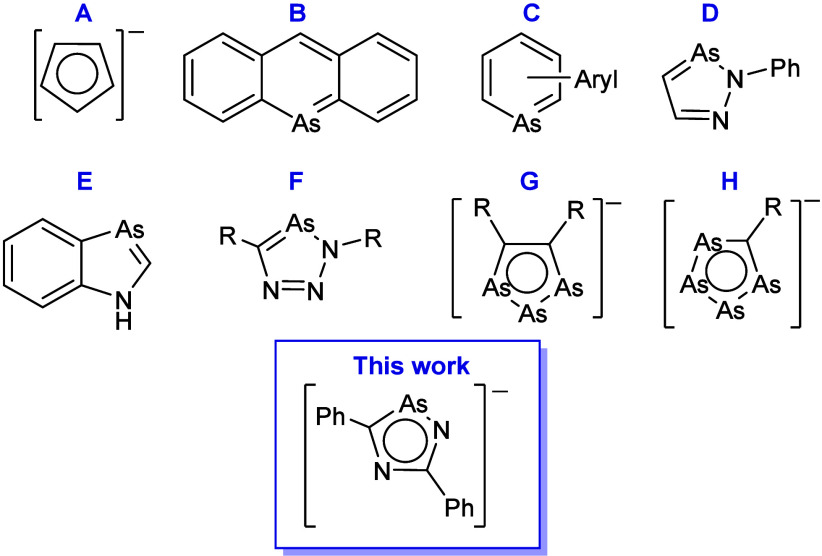
Parent cyclopentadienyl anion, selected literature examples of arsenic heterocycles, and this work.

Cyclopentadienyl anion ([Cp]^−^; see Figure 1, structure A) ligands are ubiquitous in organometallic chemistry.^1^ One interesting quality of these ligands is their ability to “ring slip” to alter their electronic contribution to the metal. The widespread success of these ligands has led to burgeoning interest in electronically tuning this platform by incorporating different elements into the five-membered ring structure. Given the isolobal relationship between the {C–H} fragment and pnictogen atoms, pnictogen derivatives of [Cp]^−^ are of particular interest.^2^ Over the past two decades phosphorus derivatives have been heavily investigated, where these rings have been hypothesized to have lower lying π orbitals compared to [Cp]^−^.^3^ Meanwhile, those featuring heavier pnictogens are far less developed.

Although arsenic heterocycles have been less well explored than their phosphorus counterparts, examples have been reported. In 1969, Bickelhaupt and co-workers synthesized the arsenic analogue of anthracene, termed “arsaanthracene” (Figure 1, structure B) and found it to be more stable than the corresponding phosphorus species.^4^ Related arsabenzenes have also been reported by Ashe and Markl (Figure 1, structure C).^5^ Heterocycles that feature both arsenic and nitrogen have been previously detailed, with Markl and co-workers preparing the phenyl-substituted diazaarsole (Figure 1, structure D) in 1973,^6^ and Heinecke et al. spectroscopically characterizing arsaindoles (Figure 1, structure E) in 1978 by reacting methylketone phenylhydrazone with arsenic trichloride.^7^ More recently in 2016, the Müller group reported the first example of an arsatriazole molecule (Figure 1, structure F), formed from the “click” reaction of 2-azidomethylpyridine with (2,4,6-tri-*tert*-butylbenzylidyne)arsane.^8^

Previously, heptapnictogen ([Pn_7_], Pn = P, As) clusters have been reported to fragment upon reaction with organic substrates and transfer pnictogen anions.^9−12^ Most relevant to this work, reaction of [Pn_7_]^3–^ clusters with alkynes have resulted in transfer of a [Pn_3_]^−^ unit from the cluster to give the 1,2,3-tripnictolide species, with the arsenic derivative shown in Figure 1, structure G.^9,11^ Furthermore, Scheer and co-workers increased the degree of arsenic incorporation into the 5-membered ring with the synthesis of the first tetraarsolyl ligand (Figure 1, structure H) through the treatment of 2,4,6-triisopropyl benzoyl chloride with As(SiMe_3_)_3_ in the presence of CsF.^13^ It is also noteworthy that complexes that feature the all-arsenic [Cp]^−^ derivative (e.g., [L_*x*_M(η^5^-As_5_)]) have also been reported,^14^ and, in these cases, the [As_5_]^−^ ring is typically generated within the coordination sphere of the metal. Here, we find that reaction of benzyl azide (BnN_3_) with a K_3_As_7_ Zintl salt leads to fragmentation of the cluster to give a novel arsolide anion. This [Cp]-like arsenic heterocycle was characterized by nuclear magnetic resonance (NMR) spectroscopy, UV–vis spectroscopy, and single-crystal X-ray diffraction (XRD), and studied using computational chemistry. Efforts were also made to probe the subsequent coordination chemistry of this heterocycle.

## Results and Discussion

**Scheme 1:**
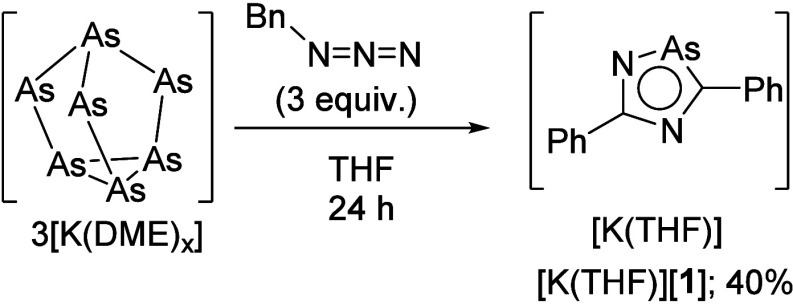
Synthesis of 1,2,4-Diazarsolide Anion [**1**]^−^

**Figure 2:**
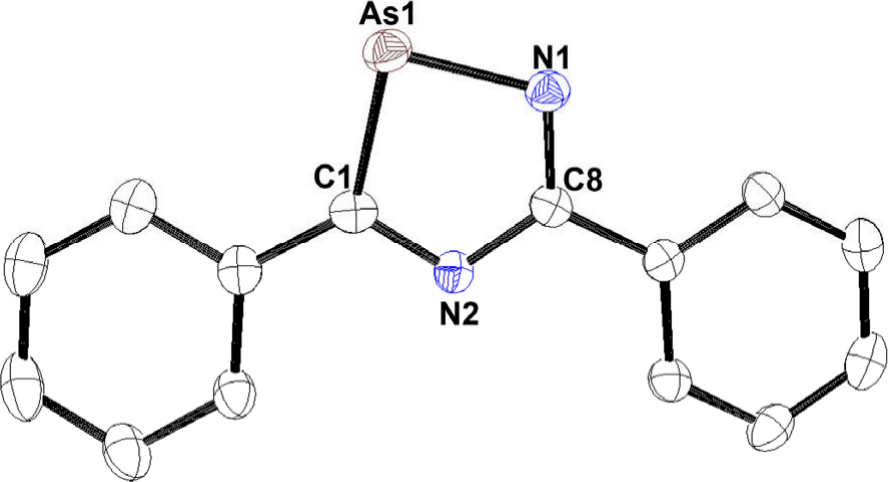
Molecular structure of [**1**]^−^ in [K(THF)][**1**]. Anisotropic displacement ellipsoids are pictured at 50% probability. Hydrogen and [K(THF)]^+^ atoms have been omitted for clarity. Legend: Arsenic (As), brown; nitrogen (N), blue; carbon (C), black.

First, [K(DME)_*x*_]_3_[As_7_] (DME = dimethoxyethane) was prepared using our literature reported method where elemental potassium and arsenic are combined.^15^ Next, a tetrahydrofuran (THF) suspension of [K(DME)_*x*_]_3_[As_7_] was treated with 3 equiv of benzyl azide (BnN_3_). Evolution of gas, presumed to be N_2_, was observed along with a color change from red-brown to an intense purple-red solution. Some precipitate was formed during the reaction, which is believed to be polyarside decomposition products. We have previously reported on polypnictogen decomposition upon reaction of related clusters with azides.^16^ Single crystals from the reaction mixture were grown through slow diffusion of hexane into a THF solution, and the product was confirmed to be the potassium salt of a 1,2,4-diazarsolide anion, [K(THF)][**1**] (Scheme 1) by single-crystal XRD studies (Figure 2). Although the mechanism for the formation of [K(THF)][**1**] is not yet understood, the [Pn_7_] clusters are known to act as a pnictogen anion source,^10^ which is hypothesized to couple with two molecules of benzyl azide which undergo C–H activation.

The solid-state structure of [K(THF)][**1**] shows the potassium coordinated to the nitrogen of three [**1**]^−^ anions with an average K–N bond distance of 2.828(18) Å, which, in turn, gives an extended chainlike structure (see Supporting Information Figures S7 and S8). Analysis of the bond metric data revealed a K–As1 bond distance of 3.836(5) Å, which is longer than previously reported potassium salts of related cyclic polyarsolide anions.^9,13,17^ This leads us to believe that K does not coordinate the As atom of [**1**]^−^ or the π-system in the solid state. The structural data also shows that the 5-membered ring is planar with one lengthened vertex when comparing to the structure of [Cp]^−^.^18^ This lengthened vertex arises from the As1–C1 (1.873(2) Å) and As1–N1 (1.845(18) Å) bonds (Figure 2). These bond lengths are in good agreement with previously reported arsoles containing one As–C and one As–N bond.^14^ A consequence of the longer bonds is the more acute C1–As1–N1 bond angle (87.00(8)°). In contrast 1,2,3-tripnictolides [Pn_3_(CPh)_2_]^−^ (Pn = P, As) possesses wider Pn–Pn–Pn angles (Pn = P: 98.95(2)°; Pn = As: 96.66(2)°) and more uniform 5-membered ring structures, similar to [Cp]^−^.^9,18^ The ^1^H NMR spectrum of [K(THF)][**1**] revealed five resonances in the aromatic region, all with equal integration. Two doublet resonances at δ = 8.3 (^3^*J*_HH_ = 7.9 Hz) and 8.0 (^3^*J*_HH_ = 6.5 Hz) ppm correspond to the *ortho*- protons on the phenyl rings, with the more downfield resonance belonging to the *ortho*-protons on the C8 phenyl ring. Two triplet resonances are also observed at δ = 7.3 and 7.2 ppm, which correspond to the *meta*- protons, and the multiplet resonance at δ = 7.1 ppm corresponds to the *para*- protons. The ^13^C{^1^H} NMR spectrum was less diagnostic, only showing aromatic singlet resonances; however, these could be assigned through 2D NMR experiments (see Section 2.1 in the Supporting Information). Anion [**1**]^−^ was also detected by negative mode mass spectrometry ([**1**]^−^: *m*/*z* = 281.0075).

**Figure 3:**
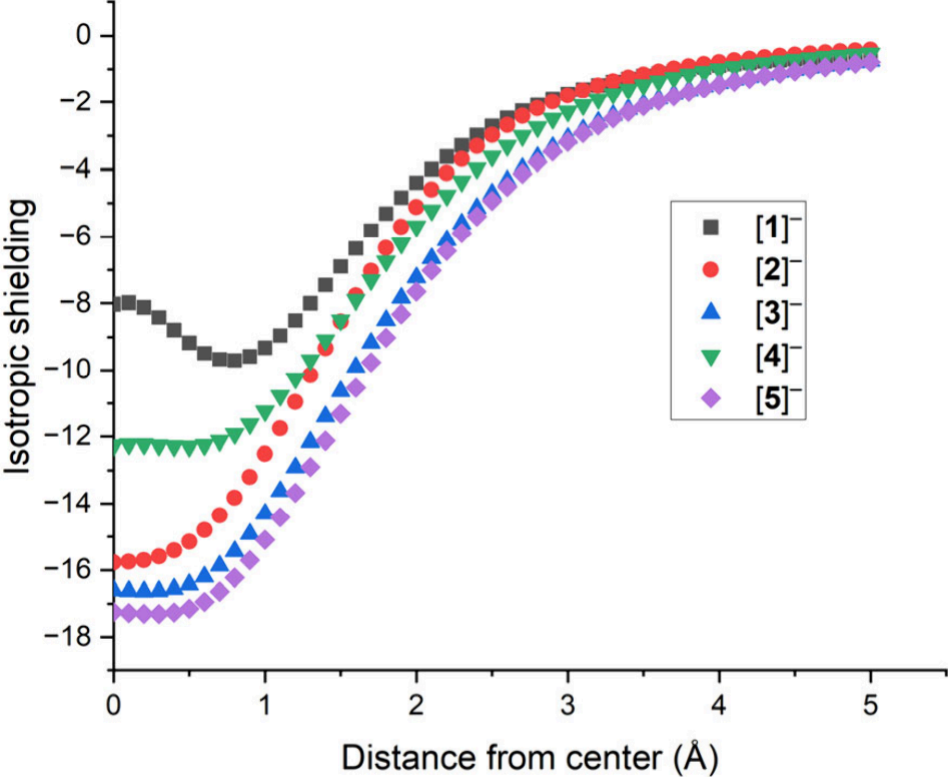
Isotropic shielding of Bq for [**1**]^−^, [**2**]^−^, [**3**]^−^, [**4**]^−^, and [**5**]^−^ between 0 and 5 Å above the plane of the ring, calculated at the pbe1pbe/6-311G(d,p) level of theory.

To assess the aromaticity of [**1**]^−^ and to allow for direct comparisons of the ring current with [Cp]^−^ and other pnictogen [Cp]-like rings, Nucleus Independent Chemical Shift (NICS) calculations were performed on [**1**]^−^, [Cp]^−^ ([**2**]^−^), [P_3_(CH)_2_]^−^ ([**3**]^−^), [P_3_(CPh)_2_]^−^ ([**4**]^−^), and [As_3_(CH)_2_]^−^ ([**5**]^−^) at the pbe1pbe/6-311G(d,p) level of theory. NICS calculations are a valuable tool in determining the (anti)aromaticity of cyclic systems by measuring the absolute isotropic magnetic shielding of a “ghost-atom” (Bq) at the center of a ring.^19^ Usually, negative NICS values indicate aromaticity, while positive values indicate antiaromaticity. The NICS(0), NICS(1) and NICS(2) correspond to the absolute magnetic shielding of Bq at the center of the ring, 1 and 2 Å above the plane of the ring, respectively. These values are reported in Table 1, with the overall NICS profile of the five species displayed in Figure 3. The NICS profile of [Cp]^−^ ([**2**]^−^), [P_3_(CH)_2_]^−^ ([**3**]^−^) and [As_3_(CH)_2_]^−^ ([**5**]^−^) are very similar, with the two 1,2,3-tripnictolides showing a greater degree of isotropic shielding of Bq close to the ring center. The two species containing phenyl substituents on the ring, [P_3_(CPh)_2_]^−^ ([**4**]^−^) and [**1**]^−^ show significantly less isotropic shielding of Bq close to the ring center, perhaps due to the delocalization of the π-system throughout the whole molecule. However, it is noteworthy that anion [**1**]^−^ was calculated to be significantly less aromatic than the other ring systems investigated. The decrease in the isotropic shielding value of Bq in [**1**]^−^ between 0 and 1 Å could be attributed to the nonuniformity of the π-system of [**1**]^−^ and indicates that the majority of the π-electron density is localized at ∼0.8 Å above the ring plane.

**Table 1:**
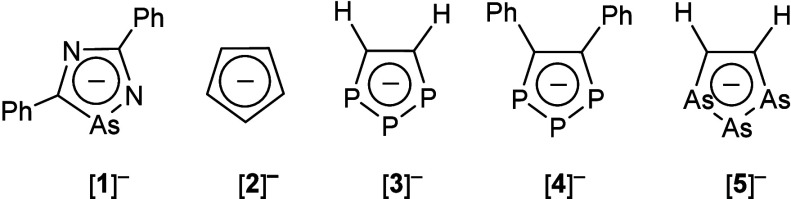
NICS Values at the Ring Center (NICS(0)) and 1 Å (NICS(1)) and 2 Å (NICS(2)) above the Ring Plane

compound	NICS(0)	NICS(1)	NICS(2)
[**1**]^−^	–8.02	–9.33	–4.40
[**2**]^−^	–15.77	–12.51	–5.12
[**3**]^−^	–16.60	–14.30	–7.22
[**4**]^−^	–12.25	–11.23	–5.69
[**5**]^−^	–17.24	–15.10	–7.65

**Figure 4:**
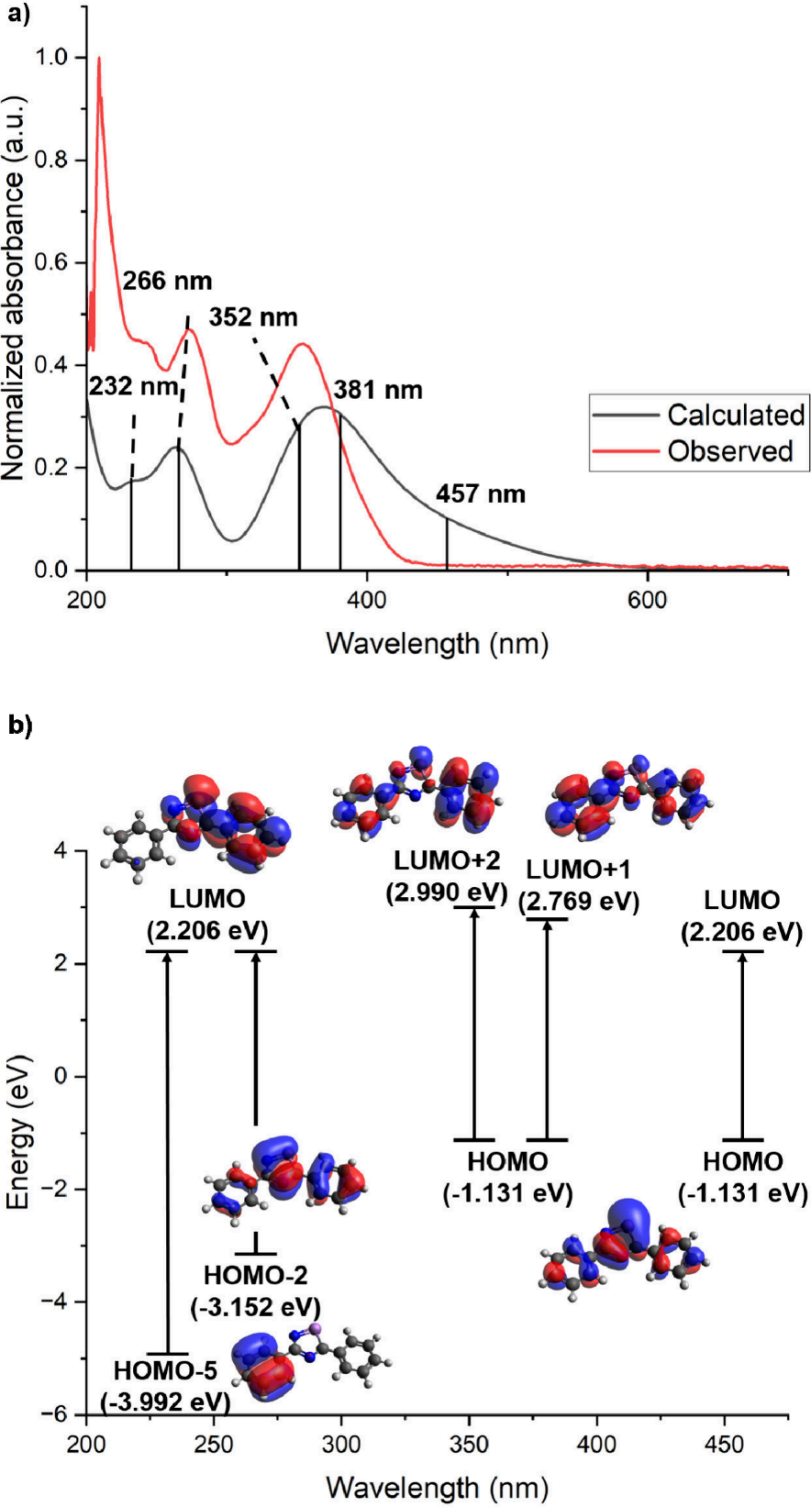
(a) UV-vis electronic absorption spectrum of [**1**]^−^ (10 μM) in THF. The exact wavelength of each calculated excitation is shown as a vertical line. (b) Excitation diagram of the possible transitions occurring in [**1**]^−^ under UV–visible light. Calculated at the pbe1pbe/6-311G(d,p) level of theory.

Compound [K(THF)][**1**] was also investigated by UV-vis electronic absorption spectroscopy, and to identify excited states and electronic transitions, time-dependent density functional theory (TD-DFT) was used. Figure 4 shows the observed UV-vis data (red trace) and the calculated transitions (black lines), both of which are in good agreement with the simulated spectral features shifted by ∼15 nm. The experimentally observed absorption features at 270 and 357 nm were found to have molar absorption coefficients of ε = 55 460 M cm^–1^ and 52 205 M cm^–1^, respectively, consistent with charge transfer.^20^ While the observed trace shows two broad signals, the computationally calculated trace suggests that the signal centered at ∼240 nm consists of two excitation events, while the signal centered at ∼340 nm is comprised of three excitation events. Analysis of the excited states from the TD-DFT calculations revealed that the transition calculated at 232 nm appears to partially be phenyl substituent to metalloid charge transfer, while the others are more delocalized intraligand charge transfers.

**Scheme 2:**
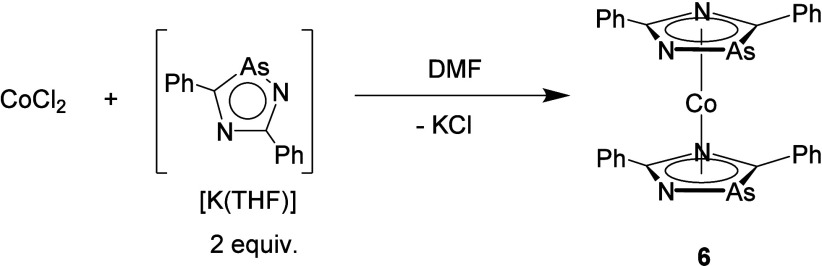
Reaction of CoCl_2_ with [K(THF)][**1**]

Finally, efforts were made to investigate the coordination chemistry of [K(THF)][**1**]. When CoCl_2_ was treated with 2 equiv of [K(THF)][**1**] in dimethylformamide (DMF), a color change to deep green was observed and the presence of a *m*/*z* peak close to the postulated sandwich complex **6** was observed by mass spectrometry (*m*/*z* = 620.6085 [M]^+^) (see Scheme 2). It is noteworthy that cobaltocene ([Cp]_2_Co) can be prepared in an analogous fashion via salt elimination.^21^ However, all efforts to cleanly isolate and fully characterize the resulting complex have thus far been unsuccessful.

To summarize, a novel arsenic-containing [Cp]-like heterocycle ([**1**]^−^) is synthesized by reacting K_3_As_7_ with BnN_3_. The structure of [**1**]^−^ was validated by NMR spectroscopy and XRD studies. The electronic structure was probed by the density functional theory and UV-vis spectroscopy. Mass spectrometry studies revealed that coordination of [**1**]^−^ to cobalt may be possible, and further investigations into the coordination chemistry of this anion are currently ongoing.

## Supporting Information Available

General information, experimental procedures, characterization data, and computational details (PDF)
Geometry-optimized coordinates (XYZ)

## Supplementary Material

om4c00476_si_001.pdf

om4c00476_si_002.xyz
