# Supplementary material for: Synthesis and Characterization of a 1,2,4-Diazarsolide Anion
Source: Organometallics. 2024 Dec 30;44(1):14–8. doi: 10.1021/acs.organomet.4c00476 (PMC11734114; doi:10.1021/acs.organomet.4c00476)
Supplement: Supplementary file 1 — om4c00476_si_001.pdf [file om4c00476_si_001.pdf]

**Synthesis and Characterization of a 1,2,4-Diazarsolide Anion**

**Supporting information**

William D. Jobbins[a], Bono van IJzendoorn[b], Meera Mehta[b]*

[a] Department of Chemistry, University of Manchester, Oxford Rd, Manchester, M13 9PL.

[b] Department of Chemistry, University of Oxford, 12 Mansfield Road, Oxford, OX1 3TA.

*meera.mehta@chem.ox.ac.uk

**Table of Contents**

**1. Methods and materials ....... S2**

1.1. Experimental considerations ....... S2

1.2. Analytical considerations ....... S2

1.3. X-ray diffraction studies ....... S3

1.4. General computational considerations ....... S3

**2. Synthesis and Characterization data ....... S4**

2.1. Synthesis of [K(THF)][AsN(CPh)N(CPh)] ([K(THF)][1]) ....... S4

2.2. Attempted synthesis of Co([1])$_2$ ....... S9

**3. Computational studies ....... S11**

3.1. Nucleus Independent Chemical Shift (NICS) calculations ....... S11

3.2. Time Dependent Density Functional Theory (TD-DFT) ....... S14

**4. Crystallography tables ....... S16**

**5. References ....... S16**

## 1. Methods and materials

### 1.1. Experimental considerations

All manipulations were carried out under a nitrogen atmosphere using standard Schlenk-line and glovebox methodology. All glassware was flame-dried before use.

Innovative Technologies anhydrous engineering solvent purification system was used to obtain dry THF, and hexane. Solvents obtained in this way were subsequently degassed. Dimethylformamide (DMF) and THF-$d_8$ were dried over 3 Å molecular sieves. All solvents were stored over activated 3 Å molecular sieves.

Potassium metal (Sigma-Aldrich), elemental grey arsenic (Alfa-Aesar), benzyl azide ($BnN_3$) (Fluorochem), and $CoCl_2$ (Thermo-Fisher) (Apollo-Scientific) were purchased from their respective vendors and used without further purification. $[K(DME)_x]_3[As_7]$ was prepared following our literature reported protocol.[1]

### 1.2. Analytical considerations

**NMR spectroscopy –** $^1H$ and $^{13}C\{^1H\}$ NMR spectra were recorded on a Bruker AVIII 400 spectrometer (operating frequencies: 399.78 MHz and 100.53 MHz for $^1H$ and $^{13}C\{^1H\}$ respectively. $^1H$ and $^{13}C\{^1H\}$ NMR chemical shifts were internally referenced to the residual THF-$d_8$ solvent resonance (THF-$d_8$: $^1H$ $\delta$ = 3.62, 1.79 ppm, $^{13}C\{^1H\}$ $\delta$ = 68.03, 26.19 ppm). NMR samples were prepared under a nitrogen atmosphere in a 5 mm J Young NMR tube.

**Elemental analysis –** Elemental analysis was carried out by the microanalysis service at the University of Manchester using a Flash 2000 elemental analyser. The average data over multiple runs is presented as data within ±0.5% could not be obtained. Melen and co-workers have found that random error can lead to discrepancies greater than ±0.4%.[2]

**Mass spectrometry –** Mass spectrometry was carried out by the mass spectrometry service at the University of Manchester using an electrospray ionization equipped Thermo Orbitrap Executive Plus Extended Mass Range mass spectrometer. Mass spectrometry samples were

prepared under an inert nitrogen atmosphere and injected directly into the ionization source of the spectrometer.

**UV-Vis spectroscopy –** UV-Vis electronic absorption spectra were recorded on a Mettler Toledo UVvisBio spectrophotometer. Samples were prepared in a J Young cuvette with path length l = 1 cm.

### 1.3. X-ray diffraction studies

**Data collection:** X-ray diffraction (XRD) data was collected for compounds [K(THF)][**1**] on a dual source Rigaku FR-X rotating anode at 100 K with Cu-Kα (1.54184 Å) radiation, equipped with a Hypix000HE detector and Oxford cryosystem. X-ray data was collected using CrysAlisPro software.

**Crystal structure determination and refinements:** X-ray data was processed and reduced using CrysAlisPro. Absorption correction was performed using empirical methods (SCALE3 ABSPACK) based upon symmetry-equivalent reflections combined with measurements at different azimuthal angles. The crystal structure was solved and refined against all $F^2$ values using the SHELX and Olex2 suite of programmes.[3] All non-hydrogen atoms were refined anisotropically. Hydrogen atoms were placed in calculated positions and refined using idealized geometries and assigned coupled isotropic displacement parameters.

### 1.4. General computational considerations

Density Functional Theory (DFT) and time-dependent DFT (TD-DFT) calculations were performed with the Gaussian 09 program package 3 (version g09, rev.d01). Geometry optimisation, UV-Vis excitations and NICS calculations were performed at the pbe1pbe/6-311G(d,p) level of theory.[4] No symmetry constraints were applied during optimisation. All minima were confirmed by the absence of imaginary frequencies. Initial geometries were prepared using X-ray diffraction coordinates where available and Facio V22.1.1.64 software. HOMO and LUMO diagrams were prepared using the Avogadro V1.2.0 software.

## 2. Synthesis and Characterization data

### 2.1. Synthesis of [K(THF)][AsN(CPh)N(CPh)] ([K(THF)][1])

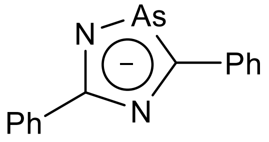

[K(THF)]$^+$

A J Young ampoule was loaded with a stir bar and $[K(DME)_x]_3[As_7]$ (100 mg, 0.16 mmol, 1 equiv.), which was suspended in THF (3 mL). A separate J Young ampoule was loaded with benzyl azide ($BnN_3$) (62 mg, 0.47 mmol, 3 equiv.) and dissolved in THF (1 mL). The THF solution of $BnN_3$ was added dropwise to the red THF suspension of $[K(DME)_x]_3[As_7]$ over a period of 5 minutes with the pressure relief system in operation. Gas evolution was observed throughout this addition. After stirring for one hour, a deep purple/red solution was observed. The reaction was allowed to stir for 12 hours in total, after which the reaction mixture was filtered and solvent removed from the filtrate under a reduced pressure to give a deep red oil. The red oil was dissolved in a minimal amount of THF and slow diffusion of hexane into this solution yielded crystals suitable for single crystal X-ray diffraction (XRD) analysis.

**Isolated yield:** 20 mg, 40% for [K(THF)][**1**]

**$^1$H NMR (400 MHZ, 298 K, THF-$d_8$):** $\delta$ = 8.33 (d, $^3J_{HH}$ = 6.8 Hz, 2H, *ortho*-H N(C*Ph*)N), 8.00 (d, $^3J_{HH}$ = 8.4 Hz, 2H, *ortho*-H As(C*Ph*)N), 7.25 (t, $^3J_{HH}$ = 7.7 Hz, 2H, *meta*-H N(C*Ph*)N), 7.17 (t, $^3J_{HH}$ = 7.7 Hz, 2H, *meta*-H As(C*Ph*)N), 7.04 (m, 2H, *para*-H) ppm.

**$^{13}$C{$^1$H} NMR (101 MHz, 298 K, THF-$d_8$):** $\delta$ = 204.5 (s, As(*C*Ph)N), 165.5 (s, N(*C*Ph)N), 144.5 (s, *quaternary-C* As(C*Ph*)N), 141.5 (s, *quaternary-C* N(C*Ph*)N), 128.6 (s, *meta-C* As(C*Ph*)N), 128.4 (s, *meta-C* N(C*Ph*)N), 127.9 (s, *ortho-C* N(C*Ph*)N), 126.0 (s, *ortho-C* As(C*Ph*)N), 125.9 (s, *para-C*), 125.6 (s, *para-C*) ppm.

**UV-Vis ($\lambda_{max}$ nm ($\varepsilon$ M$^{-1}$ cm$^{-1}$), 0.1 mM THF solution):** 355 nm ($\varepsilon$ = 52205 M$^{-1}$ cm$^{-1}$), 275 nm ($\varepsilon$ = 55460 M$^{-1}$ cm$^{-1}$).

**Mass spectrometry (ESI neg):** For $C_{14}H_{10}N_2As$ ([**1**]$^-$) Calcd.: 281.0065; found: 281.0075.

**Elemental analysis:** For [$KC_4H_8O$][$C_{14}H_{10}N_2As$]: Calcd.: C 55.10, H 4.62, N 7.14 found: C 53.27, H 4.57, N 7.32.

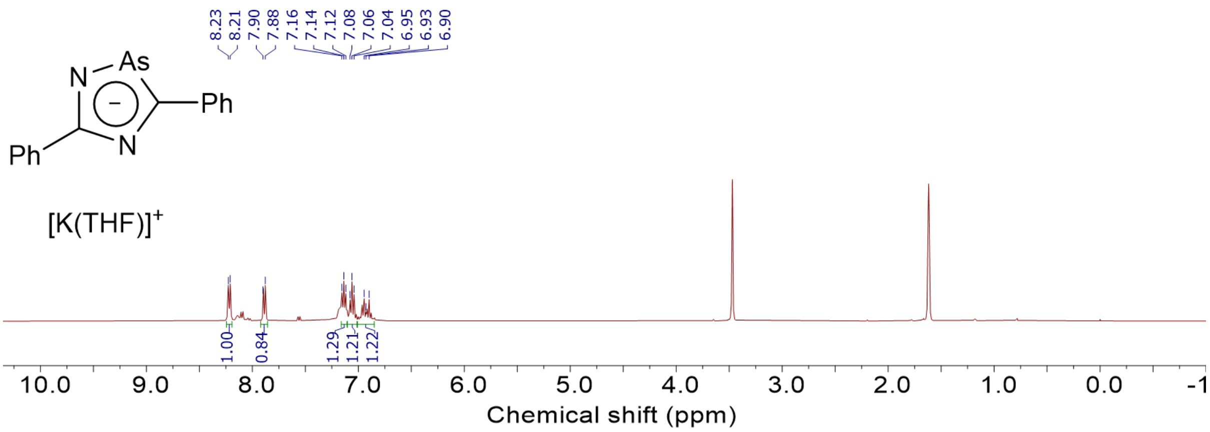


**Figure S1.** $^1H$ NMR spectrum (THF-$d_8$) of [K(THF)][**1**].

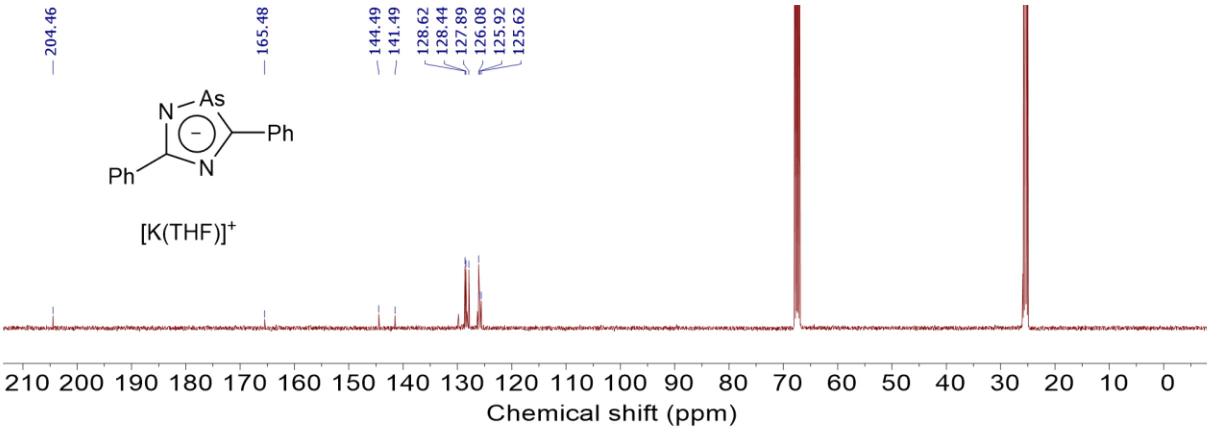


**Figure S2.** $^{13}C\{^1H\}$ NMR spectrum (THF-$d_8$) of [K(THF)][**1**].

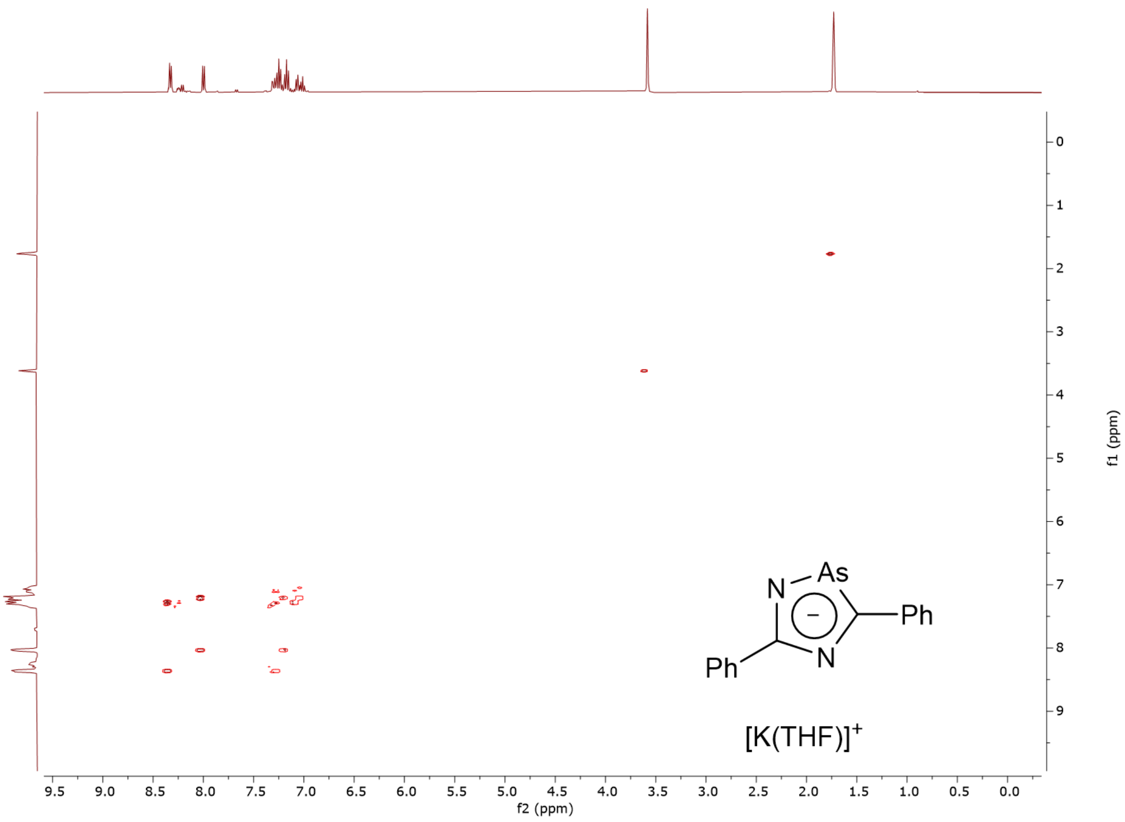


**Figure S3.** $^{1}H$ COSY NMR spectrum (THF-$d_8$) of [K(THF)][**1**].

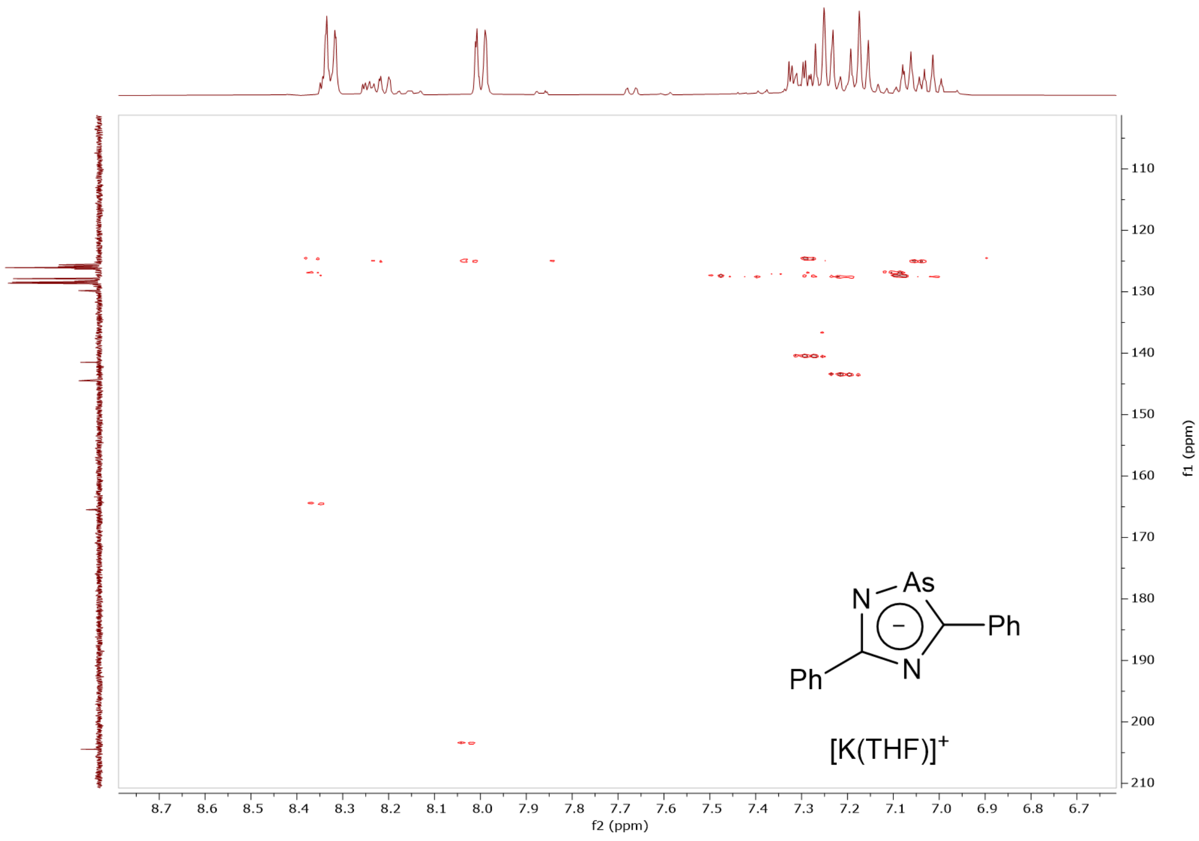


**Figure S4.** $^{1}H$ $^{13}C\{^{1}H\}$ HMBC NMR spectrum (THF-$d_8$) of [K(THF)][**1**].

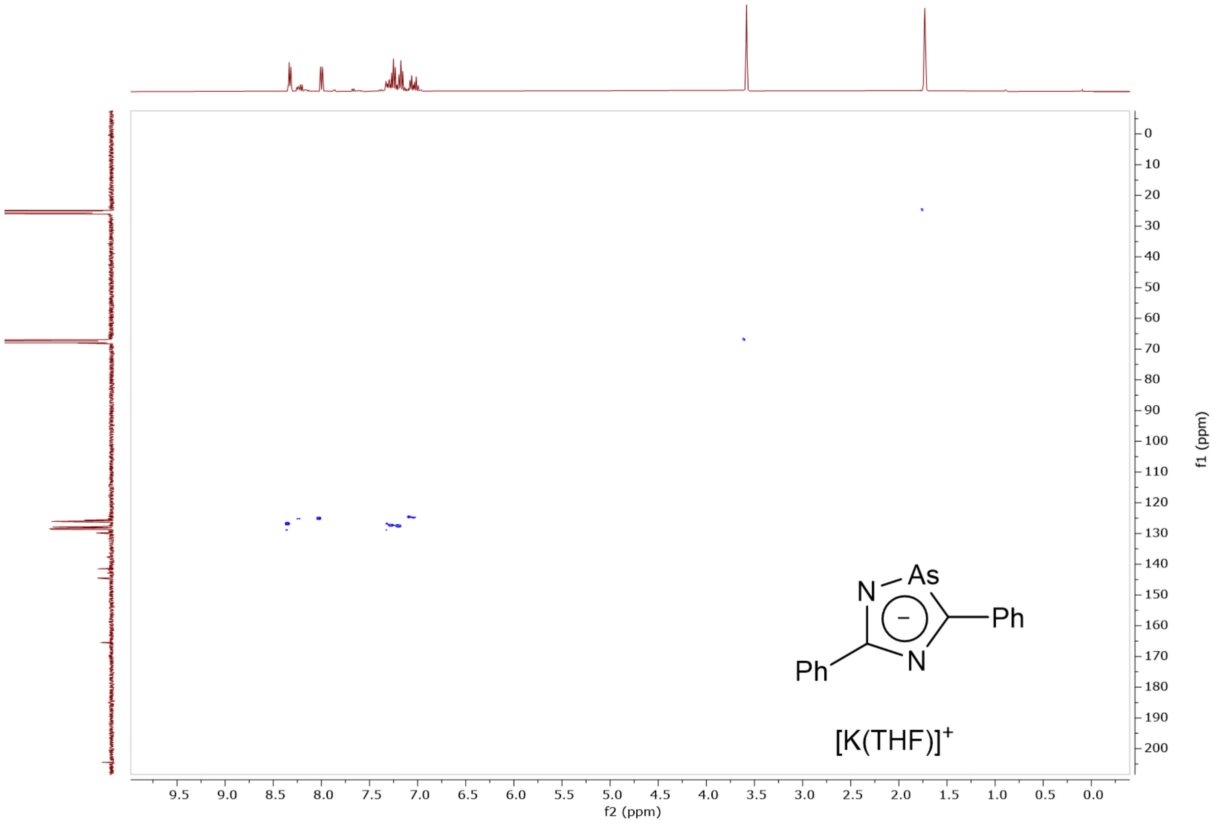


**Figure S5.** $^{1}$H $^{13}$C{$^{1}$H} HSQC NMR spectrum (THF-$d_8$) of [K(THF)][**1**].

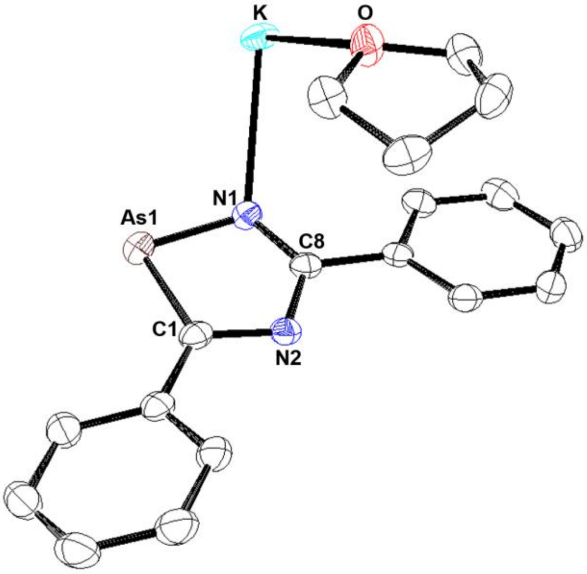


**Figure S6.** Molecular structure of [K(THF)][**1**]. Anisotropic displacement ellipsoids are captured at 50% probability. Hydrogen atoms have been omitted for clarity. Colour code: arsenic: brown; potassium: cyan; oxygen: red; nitrogen: blue; carbon: black.

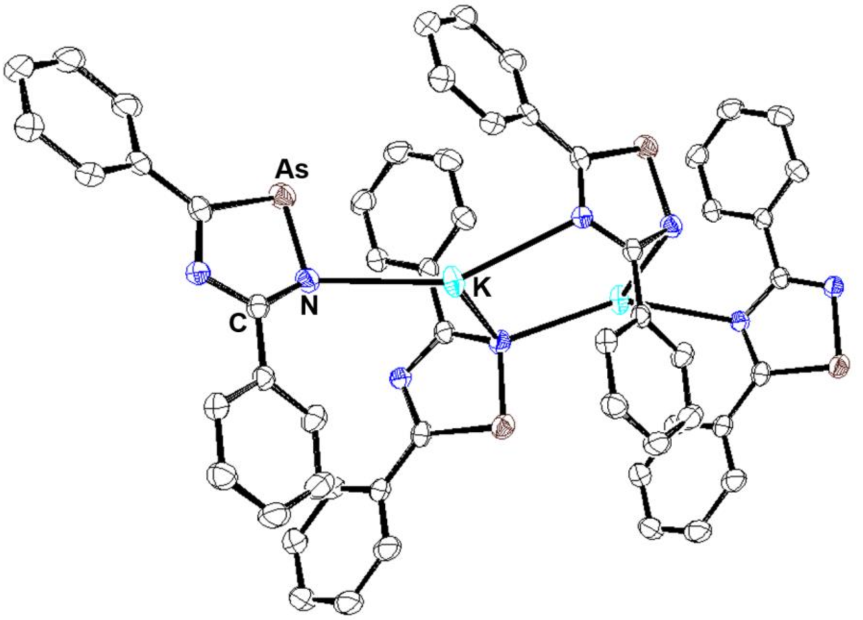


**Figure S7.** Extended molecular structure of [K(THF)][**1**]. Anisotropic displacement ellipsoids are captured at 50% probability. THF solvent molecules and Hydrogen atoms have been omitted for clarity. Colour code: arsenic: brown; potassium: cyan; nitrogen: blue; carbon: black.

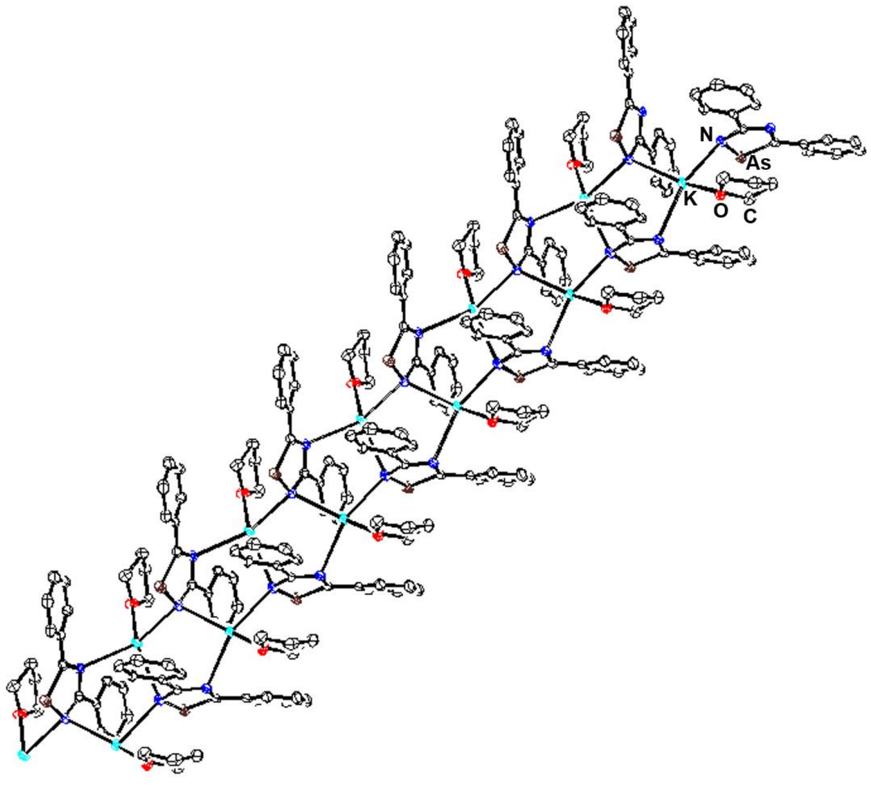


**Figure S8.** Further extended molecular structure [K(THF)][**1**]. Anisotropic displacement ellipsoids are captured at 50% probability. Hydrogen atoms have been omitted for clarity. Colour code: arsenic: brown; potassium: cyan; oxygen: red; nitrogen: blue; carbon: black.

## 2.2. Attempted synthesis of Co([1])$_2$

A J Young ampoule was loaded with a stir bar and [K(THF)][**1**] (20 mg, 0.063 mmol, 2 equiv.), dissolved in DMF (1 mL). To this solution, $CoCl_2$ (4 mg, 0.032 mmol, 1 equiv.) was added. Upon addition of $CoCl_2$ a colour change from deep red to dark turquoise was observed. The reaction was allowed to stir for 12 hours, by which point a green colour had developed. The solvent was removed under reduced pressure, affording a green residue. Multiple attempts to purify and isolate the product were made, all with no success. Some of these efforts included sublimation of the crude product under dynamic vacuum at 60 °C over 6 hours, diffusion of a layer of hexane into a THF solution of the reaction mixture, and slow evaporation of a hexane solution of the crude reaction mixture. Slow diffusion of hexane into a THF solution of the reaction mixture yielded small blue needle shaped crystals, however these were found to be unsuitable for single crystal XRD analysis. Attempts at obtaining a $^1$H NMR spectrum were made, but no resonances were observed presumably due to the presence of paramagnetic species.

**Mass spectrometry (ESI pos/neg):** For $C_{28}H_{20}As_2N_4Co$ ($[M]^+$): Calcd.: 620.9446; found: 620.6085.

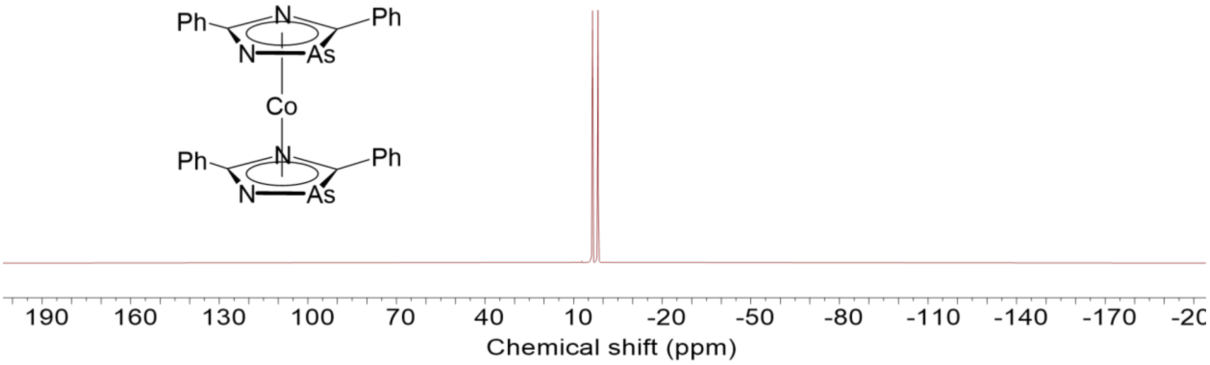


**Figure S9.** Extra-wide $^1$H NMR spectrum (THF-$d_8$) of the crude reaction of [K(THF)][**1**] with $CoCl_2$.

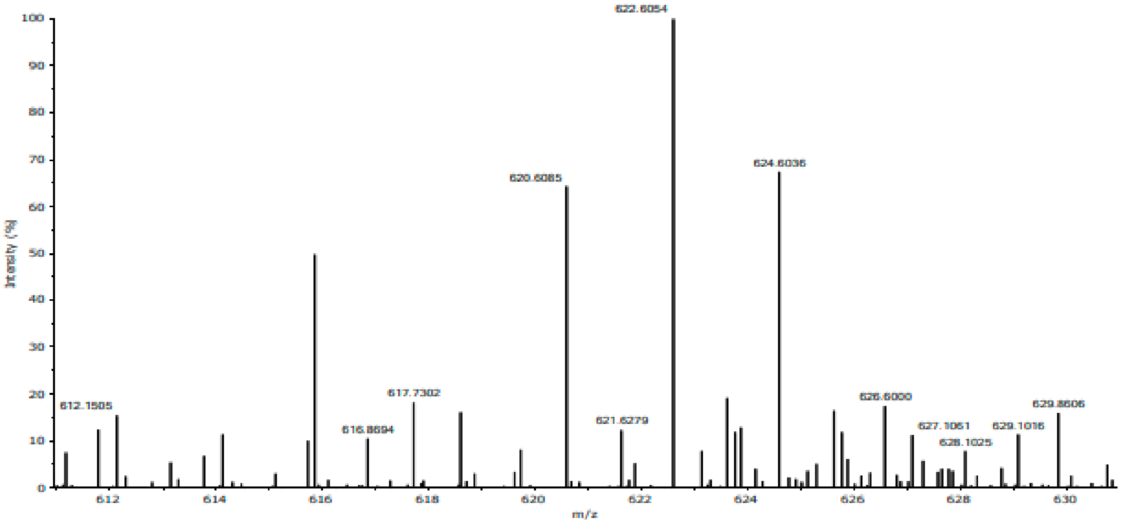


**Figure S10.** Mass spectrum of the reaction of [K(THF)][**1**] with $CoCl_2$.

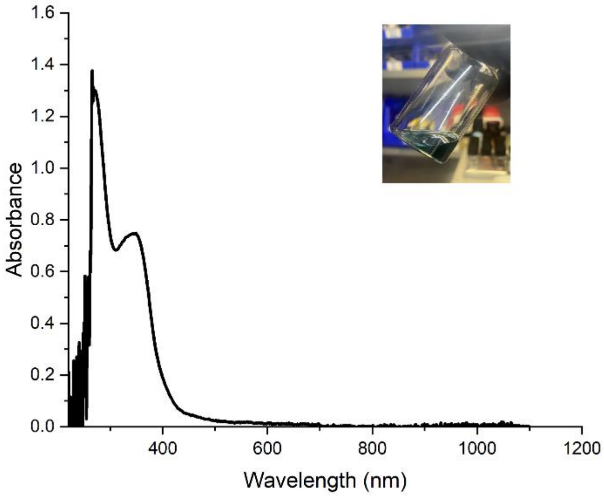


**Figure S11.** UV-Vis spectrum (DMF) of the dark turquoise solution observed with the reaction of [K(THF)][**1**] with $CoCl_2$ 10 minutes after mixing.

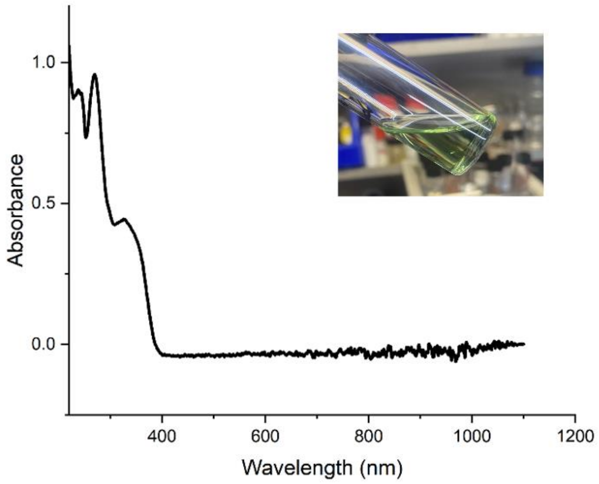


**Figure S12.** UV-Vis spectrum (DMF) of the green solution observed with the reaction of [K(THF)][**1**] with $CoCl_2$ after 12 hours.

# 3. Computational studies

## 3.1. Nucleus Independent Chemical Shift (NICS) calculations

NICS calculations were performed on [**1**]$^-$, [Cp]$^-$ ([**2**]$^-$), [$P_3(CH)_2$]$^-$ ([**3**]$^-$), [$P_3(CPh)_2$]$^-$ ([**4**]$^-$), and [$As_3(CH)_2$]$^-$ ([**5**]$^-$), by first optimizing the structures at the pbe1pbe/6-311G(d,p) level of theory and then placing "ghost atoms" (Bq) in the plane of each ring (NICS(0)) and above the plane of the ring every 0.1 Å up to 5 Å. The negative of the isotropic magnetic shielding of Bq at each point was then recorded. The results are tabulated in Table S1.

```
%nprocshared=8
%mem=16GB
%chk=RinganionNICS_Test3.chk
# nmr pbe1pbe 6-311g(d,p)
```

**Figure S13.** Example .GJF gaussian input file used in the NICS calculations.

```
#!/bin/bash --login
#$ -cwd
# Run job in directory you submitted from
#$ -pe smp.pe 8
# Run in SMP parallel environment with e.g. 8 cores

# Maximum 32

# Load g09
module load apps/binapps/gaussian/g09d01_em64t


# We now recommend using a scratch directory per job
export GAUSS_SCRDIR=/scratch/$USER/gau_temp_$JOB_ID
mkdir -p $GAUSS_SCRDIR

# Ensure correct no. of cores used. No longer done in the input file.
export
GAUSS_PDEF=$NSLOTS

$g09root/g09/g09 < RinganionNICS_Test3.gjf > RinganionNICS_Test3.log
```

**Figure S14.** Example .run Gaussian input for the NICS calculations.

**Table S1.** The isotropic magnetic shielding of $[Cp]^-$, $[P_3(CH)_2]^-$, $[P_3(CPh)_2]^-$, $[As_3(CH)_2]^-$ and **[1]**$^-$ at points between 0 and 5 Å above the plane of each ring.

| | Isotropic magnetic shielding | | | | |
|---|---|---|---|---|---|
| **Distance from centre / Å** | **[1]$^-$** | **$[Cp]^-$** | **$[P_3(CH)_2]^-$** | **$[P_3(CPh)_2]^-$** | **$[As_3(CH)_2]^-$** |
| 0 | -8.025 | -15.7675 | -16.6018 | -12.2528 | -17.2419 |
| 0.1 | -7.9759 | -15.7526 | -16.624 | -12.2205 | -17.2741 |
| 0.2 | -8.117 | -15.6976 | -16.637 | -12.2235 | -17.3017 |
| 0.3 | -8.4132 | -15.5898 | -16.6235 | -12.2503 | -17.3065 |
| 0.4 | -8.7949 | -15.4131 | -16.5607 | -12.2797 | -17.2645 |
| 0.5 | -9.1786 | -15.1534 | -16.4254 | -12.2845 | -17.151 |
| 0.6 | -9.4889 | -14.8023 | -16.1992 | -12.2383 | -16.946 |
| 0.7 | -9.6731 | -14.357 | -15.8716 | -12.1192 | -16.6371 |
| 0.8 | -9.7057 | -13.8203 | -15.441 | -11.9136 | -16.2212 |
| 0.9 | -9.5851 | -13.1995 | -14.9143 | -11.6171 | -15.7035 |
| 1 | -9.327 | -12.506 | -14.3048 | -11.2341 | -15.096 |
| 1.1 | -8.9567 | -11.7549 | -13.6298 | -10.7751 | -14.415 |
| 1.2 | -8.5027 | -10.9639 | -12.9081 | -10.2555 | -13.6788 |
| 1.3 | -7.9927 | -10.1517 | -12.1582 | -9.692 | -12.906 |
| 1.4 | -7.4512 | -9.3367 | -11.3973 | -9.1017 | -12.1139 |
| 1.5 | -6.8986 | -8.5355 | -10.6396 | -8.4999 | -11.3179 |
| 1.6 | -6.3509 | -7.762 | -9.8971 | -7.9 | -10.5309 |
| 1.7 | -5.8198 | -7.0273 | -9.1787 | -7.3124 | -9.7632 |
| 1.8 | -5.3139 | -6.339 | -8.4909 | -6.7454 | -9.0231 |
| 1.9 | -4.8387 | -5.7021 | -7.838 | -6.2044 | -8.3166 |
| 2 | -4.3975 | -5.1192 | -7.2225 | -5.6934 | -7.6479 |
| 2.1 | -3.9917 | -4.5904 | -6.6457 | -5.2142 | -7.0198 |
| 2.2 | -3.6215 | -4.1144 | -6.1077 | -4.7678 | -6.4337 |
| 2.3 | -3.2861 | -3.6888 | -5.608 | -4.3541 | -5.8902 |
| 2.4 | -2.984 | -3.3102 | -5.1457 | -3.9725 | -5.3889 |
| 2.5 | -2.7131 | -2.9745 | -4.7194 | -3.6219 | -4.9288 |
| 2.6 | -2.4713 | -2.6778 | -4.3275 | -3.301 | -4.5082 |
| 2.7 | -2.256 | -2.4158 | -3.9684 | -3.0083 | -4.1253 |
| 2.8 | -2.0649 | -2.1847 | -3.6403 | -2.7423 | -3.7778 |
| 2.9 | -1.8953 | -1.9806 | -3.3412 | -2.5011 | -3.4631 |
| 3 | -1.745 | -1.8002 | -3.0691 | -2.2831 | -3.1787 |
| 3.1 | -1.6117 | -1.6405 | -2.8221 | -2.0864 | -2.9219 |
| 3.2 | -1.4934 | -1.4988 | -2.5981 | -1.9094 | -2.6902 |
| 3.3 | -1.3881 | -1.3727 | -2.3951 | -1.7501 | -2.481 |
| 3.4 | -1.2942 | -1.2602 | -2.2112 | -1.6069 | -2.292 |
| 3.5 | -1.2102 | -1.1597 | -2.0446 | -1.4783 | -2.1212 |
| 3.6 | -1.1348 | -1.0695 | -1.8937 | -1.3626 | -1.9665 |
| 3.7 | -1.0668 | -0.9884 | -1.7567 | -1.2586 | -1.8262 |
| 3.8 | -1.0054 | -0.9152 | -1.6323 | -1.1649 | -1.6986 |

| | | | | | |
|---|---|---|---|---|---|
| 3.9 | -0.9496 | -0.8491 | -1.5191 | -1.0803 | -1.5825 |
| 4 | -0.8987 | -0.7892 | -1.416 | -1.0038 | -1.4766 |
| 4.1 | -0.8522 | -0.7348 | -1.3219 | -0.9346 | -1.3798 |
| 4.2 | -0.8095 | -0.6853 | -1.2359 | -0.8718 | -1.2912 |
| 4.3 | -0.7702 | -0.6401 | -1.1571 | -0.8146 | -1.2099 |
| 4.4 | -0.7338 | -0.5988 | -1.0848 | -0.7625 | -1.1352 |
| 4.5 | -0.7002 | -0.561 | -1.0183 | -0.7149 | -1.0664 |
| 4.6 | -0.6689 | -0.5263 | -0.9571 | -0.6713 | -1.003 |
| 4.7 | -0.6397 | -0.4943 | -0.9007 | -0.6313 | -0.9445 |
| 4.8 | -0.6125 | -0.4649 | -0.8486 | -0.5946 | -0.8903 |
| 4.9 | -0.587 | -0.4378 | -0.8004 | -0.5607 | -0.8402 |
| 5 | -0.5631 | -0.4128 | -0.7557 | -0.5295 | -0.7937 |

### 3.2. Time Dependent Density Functional Theory (TD-DFT)

TD-DFT was carried out on the pbe1pbe/6-311G(d,p) level of theory using the “nstates = 50” keyword. The anion [**1**]$^-$ was computed as the cation is expected to disassociate in solution. The calculated UV-Vis spectrum of [**1**]$^-$ was obtained and the specific wavelength of each excitation was obtained using full width at half maximum (fwhm = 2685.83 cm$^{-1}$). The calculated graph was then overlaid with the observed UV-Vis electronic absorption spectrum for [K(THF)][**1**] (10 μM) in THF for comparison. TD-DFT was also conducted on [K(THF)][**1**] with the cation present and found to be a worse match.

```
%nprocshared=8
%mem=16GB
%chk=RingTD_spc_Test3.chk
# td=(nstates=50) pbe1pbe 6-311g(d,p)
```

**Figure S15.** Example .GJF Gaussian input file for TD-DFT calculations.

```
#!/bin/bash --login
#$ -cwd
# Run job in directory you submitted from
#$ -pe smp.pe 8
# Run in SMP parallel environment with e.g. 8 cores

# Maximum 32

# Load g09
module load apps/binapps/gaussian/g09d01_em64t


# We now recommend using a scratch directory per job
export GAUSS_SCRDIR=/scratch/$USER/gau_temp_$JOB_ID
mkdir -p $GAUSS_SCRDIR

# Ensure correct no. of cores used. No longer done in the input file.
export
GAUSS_PDEF=$NSLOTS

$g09root/g09/g09 < RingTD_spc_Test3.gjf > RingTD_spc_Test3.log
```

**Figure S16.** Example .run Gaussian input file for TD-DFT calculations.

## 4. Crystallography tables

**Table S2.** Crystallography data for [K(THF)][**1**].

| | |
|---|---|
| Empirical formula | $C_{18}H_{18}AsKN_2O$ |
| Formula weight / g mol$^{-1}$ | 392.36 |
| Temperature / K | 100.01(18) |
| Crystal system | Orthorhombic |
| Space group | Pbca |
| a/ Å | 20.5621(4) |
| b/ Å | 7.27113(12) |
| c/ Å | 23.4422(4) |
| $\alpha$/° | 90 |
| $\beta$/° | 90 |
| $\gamma$/° | 90 |
| Volume / Å$^3$ | 3504.83(11) |
| Z | 8 |
| $\rho_{calc}$ g cm$^{-3}$ | 1.489 |
| $\mu$/ mm$^{-1}$ | 4.787 |
| F(000) | 1600.0 |
| Crystal size / mm$^3$ | 0.127 x 0.09 x 0.024 |
| Radiation | Cu K$\alpha$ ($\lambda$= 1.54184) |
| 2$\Theta$ range for data collection / ° | 7.542 to 151.84 |
| Index ranges | $-25 \le h \le 22$, $-8 \le k \le 6$, $-29 \le l \le 29$ |
| Reflections collected | 17451 |
| Independent reflections | 3587 [$R_{int}$ = 0.0309, $R_{sigma}$ = 0.0256] |
| Data/restraints/parameters | 3587/0/208 |
| Goodness-of-fit on F$^2$ | 1.039 |
| Final R indices [I>=2□(I)] | $R_1$ = 0.0310, $wR_2$ = 0.0866 |
| Final R indices [all data] | $R_1$ = 0.0379, $wR_2$ = 0.0908 |
| Largest diff. peak/hole/ e Å$^{-3}$ | 0.48/-0.86 |
| CCDC | 2395309 |

## 5. References

(1) Jobbins, W. D.; van IJzendoorn, B.; Vitorica-Yrezabal, I. J.; Whitehead, G. F. S.; Mehta, M. Reactivity of Tetrel Functionalized Heptapnictogen Clusters Towards Heteroallenes. *Dalton Trans.* **2023**, *52* (8), 2384–2391.

(2) Kuveke, R. E. H.; Barwise, L.; van Ingen, Y.; Vashisth, K.; Roberts, N.; Chitnis, S. S.; Dutton, J. L.; Martin, C. D.; Melen, R. L. An International Study Evaluating Elemental Analysis. *ACS Cent. Sci.* **2022**, *8* (7), 855–863.
(3) a) Sheldrick, G. M. *Acta. Cryst.* **2015**, *A71*, 3–8. b) Dolomanov, O. V.; Bourhis, L. J.; Gildea, R. J.; Howard, J. A. K.; Puschmann, H. *J. Appl. Cryst.* **2009**, *42*, 339–341.
(4) a) Hohenberg, P.; Kohn, W. Inhomogeneous Electron Gas. *Phys. Rev.* **1964**, *136* (3B), B864–B871. b) Kohn, W.; Sham, L. J. Self-Consistent Equations Including Exchange and Correlation Effects. *Phys. Rev.* **1965**, *140* (4A), A1133–A1138. c) Peng, C.; Ayala, P. Y.; Schlegel, H. B.; Frisch, M. J. Using Redundant Internal Coordinates to Optimize Equilibrium Geometries and Transition States. *J. Comp. Chem.* **1996**, *17* (1), 49–56. d) Gaussian 09, R. A., M. J. Frisch, G. W. Trucks, H. B. Schlegel, G. E. Scuseria, M. A. Robb, J. R. Cheeseman, G. Scalmani, V. Barone, G. A. Petersson, H. Nakatsuji, X. Li, M. Caricato, A. Marenich, J. Bloino, B. G. Janesko, R. Gomperts, B. Mennucci, H. P. Hratchian, J. V. Ortiz, A. F. Izmaylov, J. L. Sonnenberg, D. Williams-Young, F. Ding, F. Lipparini, F. Egidi, J. Goings, B. Peng, A. Petrone, T. Henderson, D. Ranasinghe, V. G. Zakrzewski, J. Gao, N. Rega, G. Zheng, W. Liang, M. Hada, M. Ehara, K. Toyota, R. Fukuda, J. Hasegawa, M. Ishida, T. Nakajima, Y. Honda, O. Kitao, H. Nakai, T. Vreven, K. Throssell, J. A. Montgomery, Jr., J. E. Peralta, F. Ogliaro, M. Bearpark, J. J. Heyd, E. Brothers, K. N. Kudin, V. N. Staroverov, T. Keith, R. Kobayashi, J. Normand, K. Raghavachari, A. Rendell, J. C. Burant, S. S. Iyengar, J. Tomasi, M. Cossi, J. M. Millam, M. Klene, C. Adamo, R. Cammi, J. W. Ochterski, R. L. Martin, K. Morokuma, O. Farkas, J. B. Foresman, and D. J. Fox, Gaussian, Inc., Wallingford CT, 2016.
